# Powdered coconut water (ACP 406®) as an alternative base culture medium for *in vitro* culture of goat preantral follicles enclosed in ovarian tissue

**DOI:** 10.21451/1984-3143-AR2019-0011

**Published:** 2019-11-18

**Authors:** Olga Juliana Roldan Castañeda, Francisco Léo Nascimento de Aguiar, Naiza Arcângela Ribeiro de Sá, Maria Luana Galdencio dos Santos Morais, Francielli Weber Santos Cibin, Ciro Alexandre Alves Torres, José Ricardo de Figueiredo

**Affiliations:** 1 Universidade Estadual do Ceará, Laboratório de Manipulação de Oócitos Inclusos em Folículos Ovarianos Pré-antrais, Fortaleza, CE, Brasil; 2 Universidade Federal de Viçosa, Laboratório de Fisiologia Animal e Reprodução, Viçosa, MG, Brasil; 3 Universidade Federal do Pampa, Uruguaiana, RS, Brasil

**Keywords:** ACP, base medium, goat, *in vitro* culture, preantral follicles

## Abstract

This study evaluated a powdered coconut water solution (ACP 406®) as a base culture medium on the *in vitro* survival and development of *in situ* goat preantral follicles. The ovarian fragments were either immediately fixed in Carnoy solution (non-cultured control) or individually cultured for 2 or 6 days. The following culture media (all containing 100 μg/mL penicillin and 100 μg/mL streptomycin) were evaluated: α-MEM (α-MEM alone, without additional supplementation); α-MEM+ (supplemented α-MEM); ACP (ACP®406 alone); or ACP+ (supplemented ACP®406). Additional supplementation includes: 1.25 mg/mL bovine serum albumin, 10 μg/mL insulin, 5.5 μg/mL transferrin, 5 ng/mL selenium, 2 mM glutamine, and 2 mM hypoxanthine. The endpoints (i) follicular morphology; (ii) development; (iii) estradiol production; and (iv) reactive oxygen species (ROS) were recorded. Data were analyzed using chi-square, Turkey, t-test or One-Way ANOVA. Differences were considered significant when P < 0.05. At day 2 of culture, a greater (P < 0.05) percentage of morphologically normal follicles was observed between ACP+ and ACP treatments. Moreover, at day 2 of culture, no hormonal difference (P < 0.05) was observed between ACP+ and both α-MEM treatments. At day 6 of culture when ACP and α-MEM treatments were compared the percentage of healthy follicles were similar (P > 0.05) among treatments. Overall, all treatments had lower primordial follicles (P < 0.05) accompany by greater developing follicles (P < 0.05) percentages than non-cultured control treatment, indicating primordial follicle activation. However, at day 6 of culture, the percentage of primordial follicle development were similar (P > 0.05) among the treatments. Likewise, no differences (P > 0.05) were observed for ROS production and follicular and oocyte diameters among treatments. Therefore, ACP+ has the equivalent efficiency to MEM+ in maintaining the survival and development of goat preantral follicles, representing an alternative plant-based low-cost culture medium for *in vitro* culture.

## Introduction

The vast majority (≈ 90%) of oocytes in the ovary are enclosed in preantral follicles (PAFs), which can be potentially used for the multiplication of superior genetic animals, endangered species, and infertility treatments in human (Santos et al., 2010). Therefore, one of the chief emerging strategies to avoid follicular atresia is the use of assisted reproductive technique named *in vitro* follicle culture (IVFC). In short, the goals of this technique are to rescue PAFs from ovary before they become atretic and culture them up to maturation stages to prevent follicular atresia. Overall, the efficiency of IVFC has been evaluated using several endpoints, including percentage of normal follicles, follicle and oocyte diameters, antral formation, gene expression of key factors related to folliculogenesis control, oocyte maturation, embryo rates, hormone production, reactive oxygen species (ROS) production, and so forth (for review, see Silva et al., 2016).

Even though several studies have demonstrated the efficiency of IVFC to assure the survivability and development of PAFs, the results have been limited to the *in vitro* production of a few matured oocytes in non-human primate (Xu et al., 2011), and embryos in some species, such as murine (Xu et al., 2006; Higuchi et al., 2015), porcine (Wu et al., 2001), bubaline (Gupta et al., 2008), ovine (Arunakumari et al., 2010), and caprine (Saraiva et al., 2010; Magalhães et al., 2011).

Therefore, efforts must be made to optimize the efficiency of IVFC systems, including the search for new base culture media. Among the base culture media used for IVFC, the modified Minimal Essential Medium alpha (α-MEM) has been widely employed for the *in vitro* culture of PAFs enclosed in ovarian tissue in many species, such as caprine (Cadenas et al., 2017; Sá et al., 2017), ovine (Lunardi et al., 2015), bovine (Jimenez et al., 2016), canine (Serafim et al., 2010), and equine (Gomes et al., 2015; Aguiar et al., 2017a, b). Moreover, the α-MEM base culture medium has been widely supplemented with some crucial substances to support IVFC conditions. It is well known that the presence or absence of different supplements such bovine serum albumin (BSA), insulin–transferrin–selenium (ITS), glutamine, hypoxanthine and antibiotics could affect IVFC efficiency (Silva et al., 2004). These authors have also shown that the addition of the aforementioned substances in the culture base medium improved the survival of goat ovarian follicles enclosed in ovarian tissue compared to non-supplemented base medium. However, to the best of our knowledge, the beneficial effect of the supplementation was not tested using alternative base media, such as powdered coconut water (ACP®).

Plant-based culture media represent a low-cost alternative for IVFC, especially in tropical regions. One of this promising plant-based culture medium is the coconut water solution (CWS). Previous reports demonstrated that CWS maintained follicular morphology, and stimulated activation and development after *in vitro* culture of caprine PAFs (Silva et al., 2000; Martins et al., 2005), and ovine (Andrade et al., 2002; Costa et al., 2006), as well as increased *in vitro* maturation (IVM) of bovine oocytes (Blume et al., 1997; Cordeiro et al., 2006). Notably, the CWS is a sterile and slightly acid solution, which is rich in nutrients such as proteins, salts, carbohydrates, vitamins, growth factors (phytohormones) and different electrolytes (Yong et al., 2009). However, the biochemical properties of CWS can vary among the different species of coconut. Hence, to reduce this variability, studies were conducted to evaluate the effects of dehydration on coconut water stability (Salgueiro et al., 2002). Then, a dehydrated presentation of CWS called powdered coconut water (ACP®) was developed, which consists in a subtle and uniform dehydrated powder formulation obtained after fruit selection, the collection of the endospermic liquid and submission to a heat dehydration process. ACP has been successfully used for short-term preservation of canine preantral follicles (Lima et al., 2010) as well as IVM of canine oocytes (Silva et al., 2010). However, to the best of our knowledge, no reports have evaluated the effect of ACP406® on the IVC of caprine preantral follicles. This study aimed to assess the effect of ACP 406® as a base culture medium on the *in vitro* survival and development of goat preantral follicles enclosed in ovarian tissue. The endpoints evaluated were the following: (i) follicular survival; (ii) follicular growth; (iii) estradiol production; and (iv) reactive oxygen species (ROS) production in the spent culture media.

## Methods

### Chemicals

Unless otherwise specified, the culture media and other chemicals used in the present study were purchased from Sigma Chemical Co. (St. Louis, Mo., USA).

### Ovaries

The research protocol (#062/2017) was approved by the Ethics and Animal Production Use Committee (CEUAP) of Federal University of Viçosa (UFV), Viçosa, BH, in accordance with the law nº. 11.794 and the rules issued by the Brazilian National Council for Animal Experimentation Control (CONCEA). Ovaries (n = 8) from 4 adult mixed-breed goats were collected from a local slaughterhouse, resulting in 4 replicates. Immediately post-mortem, the surrounding fat tissue, and ligaments were stripped off. Then, each ovarian pair were washed once in 70% alcohol for 10 seconds, followed by two washes in Minimum Essential Medium (MEM) supplemented with HEPES (MEM-HEPES), penicillin (100 μg/mL) and streptomycin (100 μg/mL). After that, the ovaries were placed in tubes with the same medium and transported to the laboratory at 4 °C within one hour (Chaves et al., 2008).

### Experimental protocol

In the laboratory, the ovarian cortex of each ovarian pair was divided into 18 tissue samples (approximate size, 3 × 3 × 1 mm) under sterile conditions. From these obtained fragments two fragments were randomly taken and immediately fixed for routine histology evaluation (non-cultured control). The remaining fragments (n = 16) were individually *in vitro* cultured in a well of a 24-well culture plate, each well containing 1 mL of culture medium (pH 7.2-7.4) for 2 or 6 days at 39 °C with 5% CO_2_ in the air. The culture media tested were: 1. α-MEM (α-MEM alone without additional supplementation); 2. α-MEM+ (supplemented α-MEM); 3. ACP (Powdered coconut water - ACP®406 alone, Biotechnology, Fortaleza, Ceará, Brazil); or 4. ACP+ (supplemented ACP®406). The medium supplementation was performed by the addition of 1.25 mg/mL bovine serum albumin (BSA), ITS (10 μg/mL insulin, 5.5 μg/mL transferrin, 5 ng/mL selenium), 2 mM glutamine, 2 mM hypoxanthine. It is important to emphasize that, all tested culture media contained antibiotics (100 μg/mL penicillin and 100 μg/mL streptomycin). The whole culture medium was replaced every other day (Days 2, 4, and 6). Furthermore, one mL of the spent medium was saved for hormonal and ROS analyses, and stored at -80 °C until evaluation. After medium collection at Day 2 and Day 6 the treatments ended and the fragments were processed for histological evaluation.

### Follicle morphology and development

Follicular morphology and development of PAFs were assessed in each ovarian fragment before (non-cultured control) and after Day 2 or Day 6 of *in vitro* culture. All samples were fixed in Carnoy solution at room temperature (around 25 °C) for four hours and kept in 70% alcohol until routine histology. Afterward, the ovarian tissues were dehydrated by use of a graded series of ethanol, diaphanized with xylene, embedded in paraffin wax, and cut into serial sections of 7 μm. Every slice was mounted on glass slides, stained with periodic acid-Schiff- hematoxylin, and examined under light microscopy (Nikon, Tokyo, Japan) at 400 x magnification.

Preantral follicles were classified accordingly to their morphology as morphologically normal (follicle containing an intact oocyte and granulosa cells well-organized in layers without pyknotic nuclei) or degenerate (oocyte with pyknotic nuclei, retracted cytoplasm, or disorganized granulosa cells detached from the basement membrane). Only preantral follicles in which the oocyte nucleus could be observed in each section were considered. To evaluate follicular activation (i.e., the transition from primordial to growing follicles, when surrounding squamous pregranulosa cells become cuboidal and begin to proliferate), the follicular developmental categories were determined only in morphologically normal follicles (MNF), as previously described (Silva et al., 2004). Either primordial (one layer of flat granulosa cells surrounding the oocyte) or developing follicles [transitional (one layer of flattened or cuboidal granulosa cells); primary (one layer of cuboidal granulosa cells); and secondary (two or more layers of cuboidal granulosa cells)] were calculated at Day 0 (non-cultured control) and after Day 2 and Day 6 of culture from each treatment.

Besides, the follicular and oocyte diameters per follicular category (primordial, transitional, and primary) were measured only in MNF, under an epifluorescence microscope (Zeiss, Cologne, Germany) using software (Nis-Element AR 3.0). Follicle diameter was measured from one side to the other edge of the outermost layer of granulosa cells. Oocyte diameter was measured from one edge of the oocyte membrane to the other. Two perpendicular diameters were recorded for each measurement, and the average of those two values was calculated (Santos et al., 2007).

### Reactive Oxygen Species (ROS) analysis

The levels of ROS were determined in the spent culture media at Day 2 and Day 6 by a spectrofluorimetric method (Loetchutinat et al., 2005; Sá et al., 2018). Briefly, culture media for all treatments were incubated with 10 µL of 2’,7’- dihydrodichlorofluorescein diacetate (DCHF-DA; 1mM). The oxidation of DCHF-DA to fluorescent dichlorofluorescein (DCF) was measured for detection of reactive species in the medium. The intensity of fluorescence emission (FI) was recorded at 520 nm (with 480 nm excitation) for two hours after addition of DCHF-DA to the medium.

### Estradiol analysis

Concentrations of estradiol were measured from standard aliquots (200 µL) in spent culture media (Ferreira et al., 2018) at Day 2, Day 4 and Day 6 of culture. The estradiol assay performed was the enzyme-linked fluorescent assay (ELFA) according to the manufacturer’s instructions (VIDAS® Estradiol II, ref 30431 bioMérieux SA, 376 Chemin de L’Orme, 69280 Marcy-l’Etoile - France). In the end, the fluorescence intensity emission was recorded at 450 nm, and the values of the fluorescence signal were inversely proportional to the concentration of the antigen present in the samples (Anckaert et al., 2002). The analytical sensitivity of the estradiol assay was nine pg/mL (range, 9.49 to 1596.81 pg/ml), and the intra-assay coefficient of variation was 7.5%.

### Statistical analyses

Data for continuous variables (morphologically normal, follicular development and follicular and oocyte diameters) were initially submitted to Shapiro-Wilk test to evaluate the normal distribution of the residues. Data for endpoints without a normal distribution were transformed to ranks when necessary. Statistical analyses were carried out using Sigma Plot version 11.2 (Systat Software, San Jose, CA, USA). Chi-square test was used to compare the percentage of morphologically normal and developing preantral follicles among and within treatments and the results expressed as percentages. Tukey test was used to compare follicle and oocyte diameters among treatments, follicular categories, and between days of culture. Estradiol analysis was compared among treatments within the same day of culture using the t-test, whereas, for ROS analysis, One Way Analysis of Variance was used to compare the effect of treatments between days of culture. Data are presented as the mean ± SEM. In all cases, differences were considered to be significant when P < 0.05.

## Results

### Number of follicles evaluated

A total of 177 (fresh non-cultured control), 145 and 138 (α-MEM, Day 2 and Day 6), 148 and 136 (α-MEM+, Day 2 and Day 6), 141 and 132 (ACP, Day 2 and Day 6), 162 and 151 (ACP+, Day 2 and Day 6) were assessed. Altogether, 1,330 follicles were evaluated, with an average of 147.77 ± 4.72 follicles appraised per treatment.

### Follicular morphology

**Figure 1:**
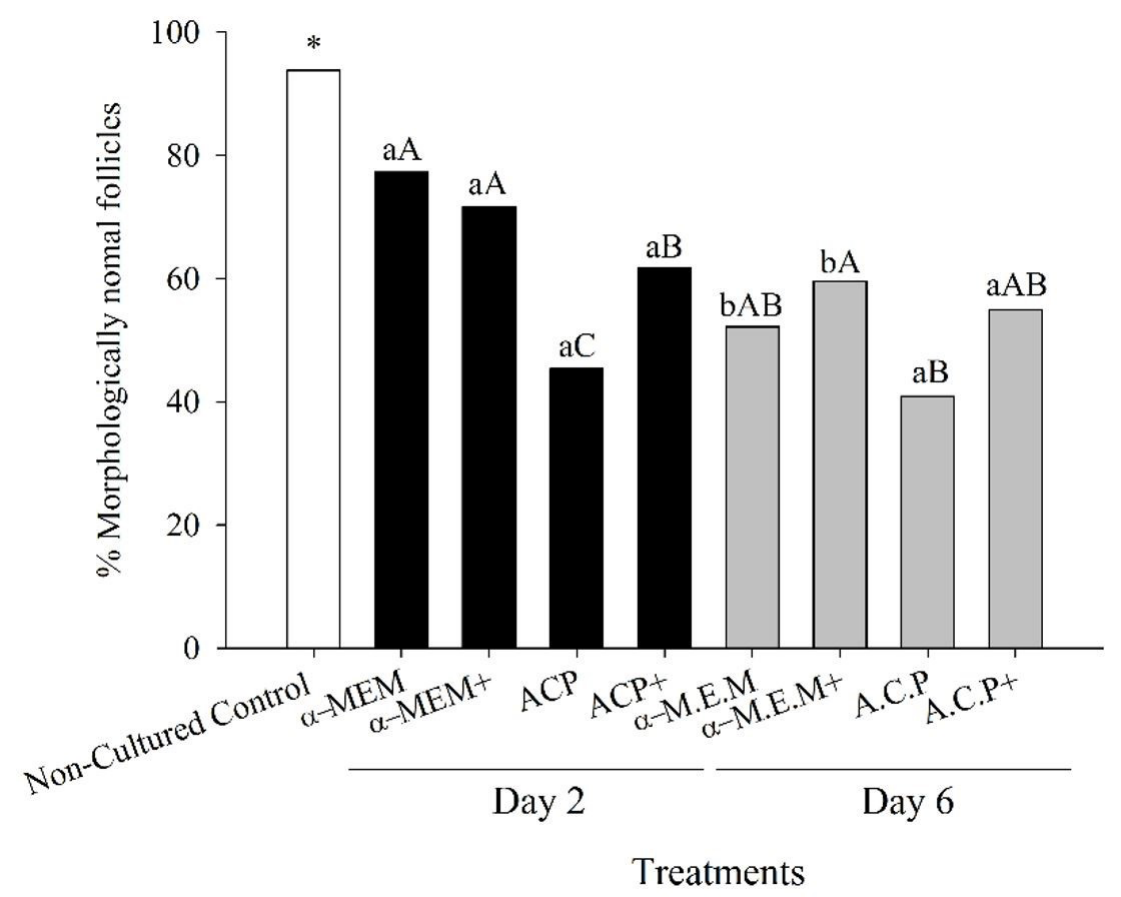
Percentage (%) of morphologically normal caprine preantral follicles in a fresh non-cultured control group (Control) and after *in vitro* culture for two (Day 2, black bars) and six (Day 6, grey bars) days in the absence (α-MEM or ACP) or presence of supplements (α-MEM+ or ACP+). *Indicates difference between non-cultured control and the other cultured treated groups. ^a,b^Within each treatment, values without a common letter differed (P < 0.05). ^A,B,C^Within days, values without a common letter differed (P < 0.05).

The percentage of MNF was reduced (P < 0.05) for all evaluated treatments after 2 and 6 days of culture when compared to the fresh non-cultured control group. Regardless the supplementation, at day 2 of culture α-MEM showed higher (P < 0.05) percentage of MNF than ACP. However, contrary to ACP and ACP+ treatments, α-MEM and α-MEM+ treatments reduced (P < 0.05) the percentage of healthy follicles from day 2 to day 6 of culture. Interestingly, at day 6 of culture when ACP and α-MEM were compared under the same condition, i.e., without or with supplementation, the percentage of normal follicles were similar (P > 0.05) (Figure 1) between the treatments.

### Follicular development

**Figure 2:**
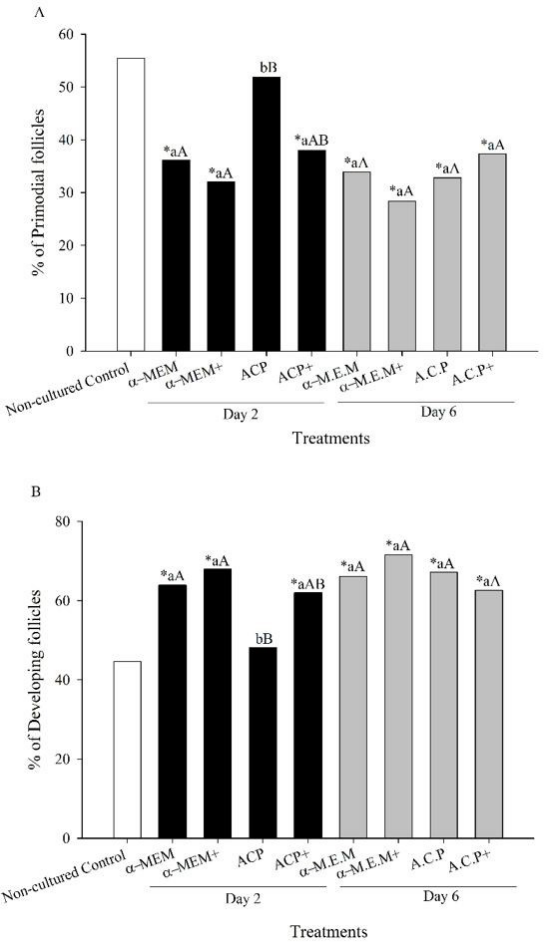
Percentage (%) of (A) primordial and (B) developing (transitional, primary and secondary) morphologically normal caprine preantral follicles in a fresh non-cultured control group (Control) and after *in vitro* culture for two (Day 2, black bars) and six (Day 6, grey bars) days in the absence (α-MEM or ACP) or presence of supplements (α-MEM^+^ or ACP^+^). *Differed (P < 0.05) from fresh non-cultured control group. ^a,b^Within each treatment, values without a common letter differed (P < 0.05). ^A,B^Within days, values without a common letter differed (P < 0.05).

The percentage of primordial and developing follicles are shown (Figures 2A and B). Compared to non-cultured control treatment, a reduction was observed (P < 0.05) in the rate of primordial along with an increase (P < 0.05) in the percentage of developing follicles after 2 days of culture, except for ACP at day 2 of culture. In addition, all treatments had greater percentage of developing follicles when compared with the non-cultured control treatment at day 6 of culture. Only for ACP treatment changes (P < 0.05) in the proportion of primordial and developing follicles were observed from day 2 to day 6 of culture. However, at day 6 of culture, similar (P < 0.05) proportion of follicular activation, i.e., the proportion of primordial and developing follicles were observed among the treatments.

### Follicular and oocyte diameter

The follicular and oocyte diameters of morphologically normal preantral follicles in primordial, intermediate and primary follicles, before (non-cultured – control) and after Day 2 and Day 6 of *in vitro* culture are shown (Table 1). When compared with Fresh non-cultured control group, all treatments had a decrease (P < 0.05) in the follicular and oocyte diameters despite the follicular category. All treatments had an increase (P < 0.05) in follicular and oocyte diameters from the primordial to primary stage. Only for the primary follicular class at Day 2 of culture, the α-MEM+D2 group had higher (P < 0.05) follicular and oocyte diameters than the ACP+D2 group.

**Table 1:** Mean (± SEM) diameters (µm) of caprine preantral follicle and oocytes (primordial, transitional, and primary) in a fresh non-cultured group and after two (D2) and six (D6) days of culture in α-MEM, or ACP in the absence or presence ^(+)^ of supplements.

	Follicle Diameter			Oocyte Diameter		
	Primordial	Transitional	Primary	Primordial	Transitional	Primary
Non-cultured control	31.0 ± 1.0 ^a^	37.5 ± 0.8 ^b^	45.7 ± 2.0 ^c^	25.4 ± 0.8 ^a^	29.2 ± 1.0 ^a^	34.4 ± 1.3 ^b^
α-MEM D2	19.7 ± 0.4 * ^aA^	23.2 ± 0.4 ^*aA^	32.9 ± 1.2 ^*bAB^	14.5 ± 0.4 ^*aA^	16.3 ± 0.5 ^*aA^	23.7 ± 1.1 ^*bBC^
α-MEM^+^ D2	20.6 ± 0.7 ^*aA^	27.1 ± 0.7 ^*bA^	33.6 ± 1.5 ^*cB^	14.9 ± 0.5 ^*aA^	19.1 ± 0.7 ^*bA^	24.2 ± 1.0 ^*cC^
ACP D2	19.3 ± 0.5 ^*aA^	25.5 ± 0.6 ^*bA^	30.8 ± 1.1 ^*cAB^	15.1 ± 0.7 ^*aA^	17.9 ± 0.5 ^*abA^	22.4 ± 0.9 ^*bABC^
ACP^+^ D2	20.2 ± 0.5 ^*aA^	24.0 ± 0.8 ^*aA^	29.3 ± 1.4 ^*bA^	15.0 ± 0.5 ^*aA^	17.2 ± 0.5 ^*abA^	19.6 ± 0.8 ^*bB^
α-MEM D6	19.3 ± 0.9 ^*aA^	26.0 ± 0.7 ^*bA^	32.0 ± 1.2 ^*cAB^	13.0 ± 0.7 ^*aA^	17.5 ± 0.6 ^*bA^	21.8 ± 1.2 ^*cABC^
α-MEM^+^ D6	20.7 ± 0.5 ^*aA^	25.0 ± 0.4 ^*bA^	30.2 ± 1.0 ^*cAB^	14.8 ± 0.6 ^*aA^	16.9 ± 0.5 ^*abA^	19.7 ± 0.9 ^*bA^
ACP D6	22.0 ± 0.6 ^*aA^	24.6 ± 0.7 ^*aA^	30.0 ± 0.7 ^*bAB^	15.0 ± 0.4 ^*aA^	16.6 ± 0.6 ^*abA^	20.0 ± 0.8 ^*bAB^
ACP^+^ D6	21.1 ± 0.6 ^*aA^	25.4 ± 0.8 ^*bA^	31.4 ± 1.0 ^*cAB^	15.9 ± 0.8 ^*aA^	17.7 ± 0.7 ^*aA^	22.1 ± 1.1 ^*bABC^

* Values differed (P < 0.05) from fresh non-cultured control group at the same follicular stage.

A,B,C Within a column, uncommon uppercase letters differed (P < 0.05).

a,b,c Within a row, uncommon lowercase letters differed (P < 0.05).

### Reactive oxygen species production

**Figure 3:**
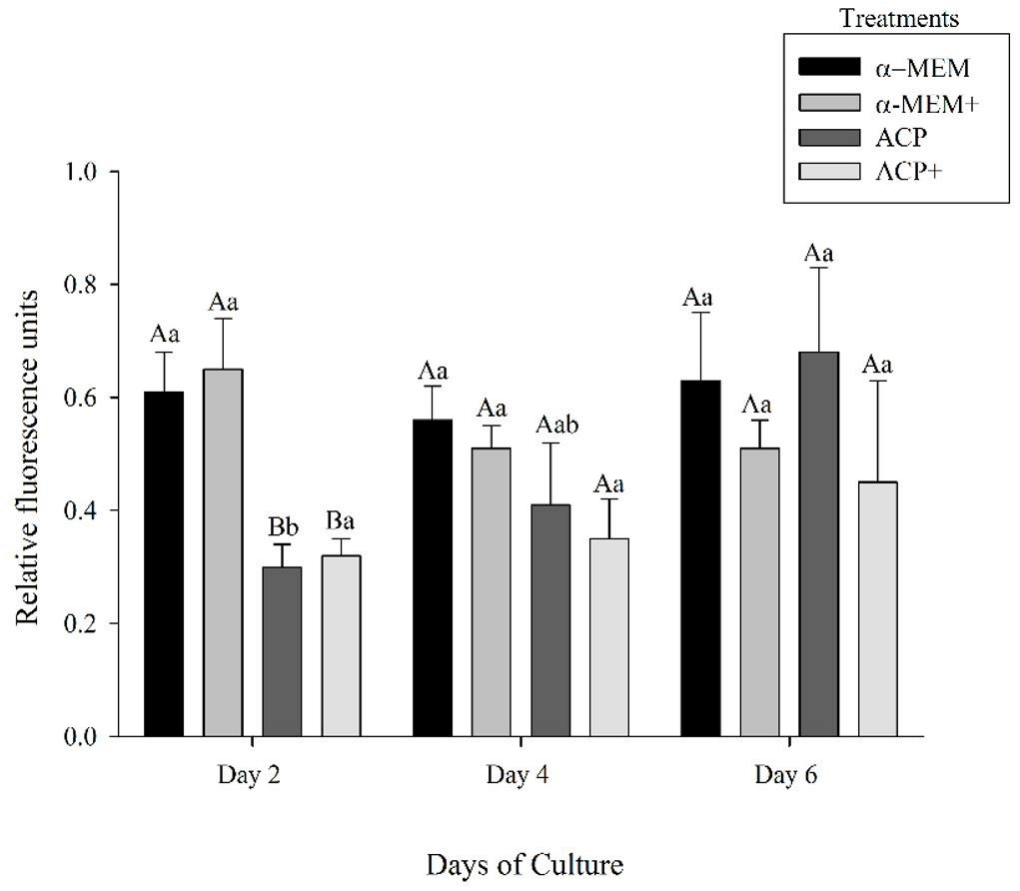
Mean (± SEM) reactive oxygen production (relative fluorescence units – microM of H_2_O_2_) produced in the medium after Day 2, Day 4, and Day 6 in the absence (α-MEM or ACP) or presence of supplements (α-MEM^+^ or ACP^+^). ^a,b^Within each treatment, values without a common letter differed (P < 0.05). ^A,B^Within days, values without a common letter differed (P < 0.05).

At Day 2 of culture, α-MEM and α-MEM+ treatments had a higher (P < 0.05) ROS production than the ACP and ACP+ (Figure 3). However, after days 4 and 6 of culture, no difference (P > 0.05) among treatments was observed. Finally, increasing (P < 0.05) in ROS production during culture was only observed in the treatment ACP without supplementation from Day 2 to Day 6 of culture.

### Estradiol production

**Figure 4:**
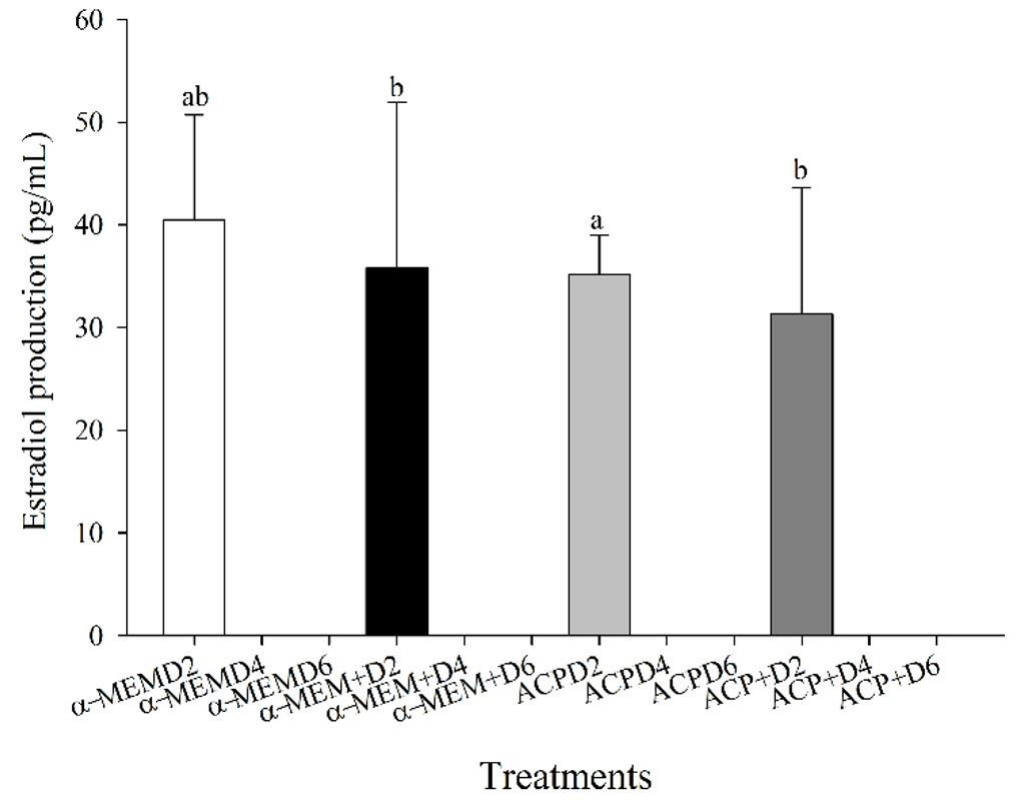
Mean (± SEM) estradiol synthesis (pg/mL; n=24) produced by caprine preantral follicles after *in vitro* culture for two (D2), four (D4), and six (D6) days in the absence (α-MEM or ACP) or presence of supplements (α-MEM^+^ or ACP^+^). ^a,b^ Values without a common letter differed (P < 0.05).

On Day 2 of culture, the ACP treatment had higher (P < 0.05) estradiol production than α-MEM+ and ACP+ treatments, but it was similar (P > 0.05) to the α-MEM group. The levels of estradiol production at Day 4 of culture was detected only in a small number (n=7) of samples (data not shown). No estradiol production was observed in any treatment after six days of culture (Figure 4).

## Discussion

To the best of our knowledge, this is the first study comparing under the same experimental condition the efficiency of two base media, ACP an α-MEM, in the maintenance of *in vitro*follicular survival and development of goat preantral follicles enclosed in ovarian tissue. Our findings demonstrated similar efficiency between ACP+ and α-MEM+ taking into account the endpoints measured at day 6 of culture.

Overall, our findings showed a decrease in the percentage of MNF in all treatments when compared with the non-cultured control. This reduction has been commonly observed in many studies using preantral follicles from different species: (Rossetto et al., 2013; Andrade et al., 2014; Gouveia et al., 2016). Even though the use of alpha modified MEM as a base culture medium showed higher survivability percentages when compared with ACP’s treatments at Day 2 of culture, such difference was no longer observed at day 6 of culture suggesting similar efficiency between the two tested base media. Such finding is probably due to the fact that both MEM (Silva et al., 2004; Peng et al., 2010) and ACP (Leong and Shui, 2002; Aroucha et al., 2005; Vigliar et al., 2006) base media are enriched formulations containing critical substances such as amino acids, vitamins, and inorganic salts.

On day 2 of culture, medium supplementation (with the addition of BSA, glutamine, hypoxanthine, and ITS) increased the percentage of MNF in the ACP medium and similar activation rate between ACP and α-MEM was only observed when ACP was supplemented. These results are in agreement with previous studies that reported the beneficial effect of the above supplements on the *in vitro* culture of caprine preantral follicles (Silva et al., 2004). Overall, at days 2 and 6 of culture in all treatments, it was observed a significant reduction in the percentage of primordial follicles with a concomitant increase in the rate of developing follicles when compared with the non-cultured control group. These results are in agreement with previous studies that reported spontaneous *in vitro* activation of primordial follicles in several species including caprine (Matos et al., 2007), murine (Eppig and O’Brien, 1996), equine (Haag et al., 2013), bovine (Tang et al., 2012), and primates (Wandji et al., 1997). In the present study, when performed culture in the presence of ACP without supplementation, primordial follicle activation was lower than the other treatments and did not differ from non-cultured control. However, this was not observed at day six showing that in the ACP treatment occurred a temporary delay in the primordial follicle activation, followed by a compensatory effect during culture. Similar findings were observed for the percentage of MNF endpoint in the present study.

Regarding follicular and oocyte diameter, all treatments reduced the follicular and oocyte diameters compared to the control group. Similar results were reported in goats (Silva et al., 2002) and mares (Aguiar et al., 2017a). Considering that follicular and oocyte diameters were only recorded using MNF, the diameter decreasing during culture is probably due to the atresia of more advance follicle stages (i.e., primary and secondary) that are more likely to undergo atresia than primordial follicles (Aguiar et al., 2016). The non-detectable levels of estradiol support this statement at the end of culture and this hormone is usually produced by late secondary follicles, as well as early antral follicles (Cecconi et al., 1999).

The assessment of ROS production is an imperative tool to determine the presence of free radicals that may lead to deleterious effects on cultured cells (Talebi et al., 2012). In fact, it was previously demonstrated that an IVFC environment with an inappropriate concentration of ROS might initiate apoptosis, provoking damages in all follicular stages (Aguiar et al., 2016; Sá et al., 2017). Our study demonstrated that after six days of culture, no difference among treatments in ROS production was observed. It is known that ACP is endowed with antioxidants such as ascorbic acid, a potent free radical scavenger, redox catalyst that can reduce and neutralize ROS, protecting cells against oxidative stress (Wang et al., 2002), as well to minimize the cost of IVC with the use of antioxidant supplementation.

In conclusion, ACP+ has the equivalent efficiency to MEM+ in maintaining the survival and development of goat preantral follicles. Additionally, no differences were observed for the ACP+ and MEM+ concerning estradiol production and ROS levels after IVFC period. However, ACP has the advantage of being a natural product at a lower-cost and may be an alternative medium for *in vitro* follicle culture.
